# No difference in effectiveness of treatment simplification to boosted or unboosted atazanavir plus lamivudine in virologically suppressed in HIV-1-infected patients

**DOI:** 10.1371/journal.pone.0203452

**Published:** 2018-09-20

**Authors:** Alicia Gutierrez-Valencia, Coral García, Pompeyo Viciana, Yusnelkis Milanés-Guisado, Tamara Fernandez-Magdaleno, Nuria Espinosa, Juan Pasquau, Luis Fernando López-Cortés

**Affiliations:** 1 Unidad Clínica de Enfermedades Infecciosas, Microbiología y Medicina Preventiva, Instituto de Biomedicina de Sevilla/Hospital Universitario Virgen del Rocío/CSIC/Universidad de Sevilla, Sevilla, Spain; 2 Servicio de Enfermedades Infecciosas, Hospital Universitario Virgen de las Nieves, Granada, Spain; University of KwaZulu-Natal, SOUTH AFRICA

## Abstract

**Background:**

Simplification strategies of antiretroviral treatment represent effective tools for the reduction of drug-induced toxicity, resistance mutations in case of virological failure and costs.

**Objectives:**

To assess the effectiveness of simplification to atazanavir/ritonavir (ATV_rtv_) or unboosted atazanavir (ATV_400_) plus lamivudine, and if low plasma or intracellular ATV C_trough_ influence virological outcomes.

**Methods:**

Ambispective observational study in patients with undetectable HIV-RNA who were switched to ATV_rtv_ or ATV_400_ plus lamivudine once daily. Previous virological failures (VF) were allowed if the resistance tests showed major resistance mutation neither to ATV nor to lamivudine. VF was defined as two consecutive plasma HIV-RNA >200 copies/mL. Effectiveness was assessed by intention-to-treat and on-treatment analyses. Plasma and intracellular ATV C_trough_ were measured by LC-MS/MS.

**Result:**

A total of 246 patients were included. At week 48, the Kaplan–Meier estimation of efficacy within the ATV_rtv_ and ATV_400_ groups were 85.9% [95% confidence interval, (CI_95_), 80.3–91.4%] versus 87.6% (CI_95_, 80.1–94.1%) by intention-to-treat analysis (p = 0.684), and 97.7% (CI_95_, 95.2–100%) versus 98.8% (CI_95_, 97.0–100%) by on-treatment analysis (p = 0.546), respectively. Plasma and intracellular C_trough_ were significantly higher with ATV_rtv_ than with ATV_400_ (geometric mean (GM), 318.3 vs. 605.9 ng/mL; p = 0.013) and (811.3 vs. 2659.2 ng/mL; p = 0.001), respectively. Only 14 patients had plasma C_trough_ below the suggested effective concentration for ATV (150 ng/mL). No relationship between plasma or intracellular C_trough_ and VF or blips were found.

**Conclusion:**

Boosted or unboosted ATV plus lamivudine is effective and safe, and the lower plasma C_trough_ observed with ATV_400_ do not compromise the effectiveness of these simplification regimens in long-term virologically suppressed HIV-1-infected patients.

## Introduction

The first attempts of simplifying antiretroviral treatment (ART) in virologically suppressed HIV-1-infected patients were less effective compared with maintaining triple-drug therapy, probably due to the low genetic barrier and/or antiviral potency of the drugs used at that time [1,2]. In recent years, the availability of new drugs with improved genetic barrier and potency, particularly ritonavir-boosted protease inhibitors (PI), have led to a re-emergence of simplification strategies. The key rationales for simplifying ART are the reduction of both drug-induced toxicities and the risk of resistance mutations in case of virological failure, as well as the cost [3–7]. Two randomized clinical trials have demonstrated non-inferiority of ATV_rtv_ plus lamivudine (3TC) compared with ATV_rtv_ plus two nucleos(t)ide reverse transcriptase inhibitors (NRTIs) in HIV-infected patients with virological suppression (VL) [8–10]. Based in their results, dual therapy including atazanavir 300 mg plus ritonavir 100 mg (ATV_rtv_) plus 3TC might represents a good simplification strategy, as ATV has been associated with lower rates of lipid abnormalities than other PIs [11–13] and has a good resistance profile. However, ATV_rtv_ is not always well tolerated due to potential toxicity related both to high ATV plasma concentrations as well as to the use of ritonavir, including gastrointestinal disturbances, lipid profile alterations, and hyperbilirubinemia. Indeed, it has been observed that switching patients with virological suppression on ATV_rtv_ plus two NRTIs to 400 mg unboosted ATV once daily (ATV_400_) improves toxicity and tolerability without loss of virological suppression [14–18].

However, dual therapy comprising ATV_400_ plus 3TC has been rarely explored, although some data suggest similar effectiveness as compared to ATV_rtv_ plus 3TC in patients on long-lasting virological suppression [19,20].

A minimum plasma trough concentration (concentration at the end of interval dosing; C_trough_) of 150 ng/mL has been proposed for ATV to be effective when given with two NRTIs [21]. Since the pharmacokinetic variability of ritonavir-boosted ATV is high, it is not uncommon for patients to show an ATV plasma trough concentration (C_trough_) below this recommended level. In the case of ATV_400_, the plasma concentrations are lower and show an even higher variability than with ATV_rtv_ [22–24]; however, it remains unknown whether this influences the effectiveness of the drug to a higher extent than with ATV_rtv_ when administered in dual therapy.

Therefore, the aim of this study was to determine the effectiveness of boosted and unboosted ATV plus 3TC in virologically suppressed HIV-1-infected patients, as well as to evaluate the relationship between plasma and intracellular ATV C_trough_ with the virological outcome.

## Material and methods

### Study population

This ambispective observational study was carried out at two Spanish University Hospitals. All patients with virological suppression at least for one year who switched to a dual therapy with either ATV_rtv_ or ATV_400_ plus 3TC once daily from January of 2011 to May of 2014 (retrospective part) and from June 2014 to December 2015 (prospective part) were included. The reasons for switching were the presence of adverse effects (AEs) with previous regimens, drug-drug interactions and simplification to a regimen with a lower pill burden. These regimens were not prescribed in case of pregnancy, hepatitis B coinfection or concomitant use of drugs with potential interactions with ATV pharmacokinetics. Additionally, the presence of cirrhosis with clinical or analytical data of liver failure, and ≥1 major resistance mutations to ATV (I50L, I84V, and N88S) or 3TC (K65R/E/N or M184I/V) in the genotypic resistance tests lead to exclusion of the patient. The study was designed and conducted according to the principles of the Declaration of Helsinki and was approved by the Spanish Agency of Medicines and Healthcare Products and the Coordinating Committee on Ethics in Biomedical Research of Andalucía. All patients provided signed informed consent, except in those retrospective cases as, according to Spanish law, the retrospective studies do not require informed consent if only completely anonymous information from existing records was collected, thereby ensuring the protection of personal data in accordance with the Personal Data Protection Organic Law15/199 enacted on December 13, 1999.

### Endpoints, follow-ups, and assessments

The primary clinical endpoint was treatment effectiveness, measured as the percentage of patients who maintained virological suppression after 48 weeks according to intention-to-treat analysis (non-complete/missing = failure). VF was defined as a confirmed plasma HIV-RNA of >200 copies/mL, considering the time of the first assessment meeting the failure criteria as the time of failure, or a single HIV-RNA level >200 copies/mL in case of subsequent loss of follow-up. A cut-off level of 200 copies/mL was chosen since it represents a more accurate measurement of VF than lower cut-off values [25,26]. The change of ATV_rtv_ to ATV_400_ due to intolerance to ritonavir was not considered as failure. Viral blip was defined as a single HIV-RNA value >50 copies/mL without subsequent confirmation. As a secondary outcome, virological efficacy and its relationship with plasma and intracellular ATV concentrations were assessed in an on-treatment approach where patients who were lost to follow-up, voluntarily dropped, discontinued therapy due to AEs or changed the study regimen due medical decision without VF criteria were excluded from analyses.

A standard checklist was used for recording information extracted from electronic medical records, including demographic variables, clinical and laboratory data at baseline, after 1 month, and every 3 months thereafter. CD4^+^ T cell counts and plasma HIV-RNA were measured by flow cytometry and the Cobas AmpliPrep-Cobas TaqMan HIV-1 test (v 2.0. Roche Diagnostics, Basel, Switzerland; lower detection limit of 20 copies/mL), respectively. AEs were categorized via the standardized toxicity-grade scale used by the AIDS Clinical Trials Group [27]. However, in patients with chronic hepatitis C or cirrhosis, toxicity was classified according to changes relative to the baseline values rather than the upper limit of normality: grade 0, <1.25 x baseline; grade 1, (1.25 to 2.5) x baseline; grade 2, (2.6 to 3.5) x baseline; grade 3, (3.6 to 5) x baseline; and grade 4, >5 x baseline.

### Pharmacokinetic data

A pharmacokinetic study was performed in the subgroup of patients included prospectively, where at least one sample per patient was obtained during the follow-up. Blood samples for ATV C_trough_ were collected into EDTA and cell preparation tubes (CPTs; Becton Dickinson Vacutainer) at 24 ± 0.5 h after the previous dose taken after a standard breakfast (otherwise, blood samples were discarded). Within 1 h after collection, the tubes were centrifuged at 1500 g for 20 min at room temperature. Plasma was transferred to cryotubes and stored at -80° C until analysis. The cell layer from the CPT tubes was transferred to 15 mL Falcon tubes, peripheral blood mononuclear cells (PBMC) were then fast washed twice in 10 mL ice-cold 0.9% NaCl solution and centrifuged at 1500 g for 10 min at 4°C. The cell pellets were transferred to Eppendorf tubes and washed with 1.5 mL ice-cold 0.9% NaCl solution. Once centrifuged, the supernatants were aspirated and the cell pellets were weighed. Afterwards, the pellets were dissolved in 1ml extraction solution (methanol:water, 70:30, v/v), and then stored at -80°C, for no longer than 3 months, until analyses. For ATV intracellular concentrations, PBMC aliquots were weighed and their volume was calculated as volume = weight/density. Since the density of mononuclear cells is 1.077 and that of plasma 1.030, the weight of the aliquots was equalized with their volume.

Plasma and intracellular concentrations were determined by LC-MS/MS. The separation was performed on a Phenomenex Luna C18 (5 μm, 150 x 2.0 mm) analytical column. The mobile phase was composed of a 2 mM ammonium acetate 0.1% formic acid and acetonitrile 0.1% formic acid. The drugs were extracted from the blood plasma by protein precipitation, using acetonitrile containing a deuterated internal standard. The standard curves were highly linear over the range of 10 to 2000 ng/mL. The intra- and inter-assay precision and accuracy were <15% in both biological samples.

### Statistical analysis

Categorical variables were compared using the χ^2^ test or the Fisher´s exact test, while quantitative variables were analyzed using the Student´s t test or the Mann–Whitney nonparametric test, respectively, according to their distribution. The ATV C_trough_ were summarized as geometric means (GM), interquartile range (IQR), and range. The intra- and inter-subject variability in ATV C_trough_ was assessed by the coefficients of variation (CV) of all the available values from each patient throughout the follow-up period. The correlations between plasma and intracellular concentrations were assessed by Spearman’s correlation coefficients. Time-to-event analyses were performed by using Kaplan–Meier survival curves and the log rank test. Variables with p-values < 0.2 in the univariate analysis, as well as those that potentially affect the efficacy of the treatments, such as age and gender, were entered into Cox proportional hazard models. The adjusted hazard ratio (AHR) and the respective 95% confidence intervals (CI) were calculated. Statistical analyses were performed using the IBM software (SPSS v. 23.0, Chicago, USA), and p-values <0.05 were considered significant.

## Results

A total of 246 patients were included in the study, 149 on ATV_rtv_ and 97 on ATV_400_, whose baseline characteristics are summarized in Table 1. Eighty-two and seventy-eight patients were included prospectively in the ATV_rtv_ and ATV_400_ group, respectively. Baseline characteristics of the patients according to the time of inclusion are provided in S1 Table.

**Table 1:** Baseline characteristics of the study populations.

	ATV_rtv_ + 3TC n = 149	ATV_400_ + 3TC n = 97	*p*
Male, no. (%)	113 (75.8)	70 (72.2)	0.521
Age, years, M (IQR)	49 (43–53)	51 (45–56)	0.026
Weight, kg, M (IQR)	70 (62–80)	71 (65–81)	0.633
Nadir CD4^+^/μl, M (IQR)	166 (59–269)	164 (90–268)	0.354
Risk factor for HIV, no. (%)			0.228
Previous iv drug use	63 (42.3)	39 (39.2)	
Homosexual	46 (30.9)	21 (21.6)	
Heterosexual	40 (26.8)	37 (38.1)	
Chronic hepatitis C, no. (%)	55 (36.9)	43 (44.3)	0.237
Cirrhosis no. (%)	18 (12.1)	11 (11.3)	0.832
Previous months on ART, M (IQR)	91(31–185)	63 (33–118)	0.004
Months with HIV-RNA < 20 copies/mL, M (IQR)	60 (35–112)	48 (24–80)	0.333
Previous ART combinations			0.182
PI_rtv_ (+ 2 NRTIs), n (%)	83 (55.7)	63 (64.9)	
Atazanavir	73 (88.0)	61 (96.8)	
Darunavir	3 (3.6)	2 (3.2)	
Lopinavir	7 (8.4)	0 (0.0)	
NNRTI (+ 2 NRTIs), n (%)	16 (10.7)	8 (8.2)	
Efavirenz	6 (37.5)	4 (50)	
Nevirapine	1 (6.3)	1 (12.5)	
Rilpivirine	9 (56.3)	3 (37.5)	
INSTI (+ 2 NRTIs), n (%)	6 (4.0)	4 (4.1)	
Elvitegravir	1 (16.7)	1 (25.0)	
Dolutegravir	0 (0.0)	1 (25.0)	
Raltegravir	5 (83.3)	2 (50.0)	
Other PI_rtv_ regimens, n (%)	44 (29.7)	22 (22.7)	
CD4^+^/μl, M (IQR)	693 (519–918)	698 (531–953)	0.562

M (IQR), Mean (interquartile range). ATV_rtv_, atazanavir boosted with ritonavir. ATV_400_, unboosted-atazanavir. ART, antiretroviral treatment. PI_rtv_, ritonavir-boosted protease inhibitor. NRTIs, nucleoside reverse transcriptase inhibitors. INSTI, integrase strand transfer inhibitor.

The reasons for switching to dual therapy based on ATV were AEs with previous regimens (38.6%), drug-drug interactions (12.2%), and simplification (49.2%). Before switching to dual therapy, 69.5% of the patients were on an ATV_rtv_-based regimen. Sixty-one patients (25%) had experienced a previous VF while on PI-based regimens (saquinavir, 36.1%; indinavir, 32.7%; nelfinavir, 16.4%; lopinavir, 11.5% and fosamprenavir, 3.3%), but no major resistance mutations for ATV were found in the genotype resistance tests just after these VFs. Eighty-eight percent of these patients had a subsequent treatment based on ritonavir-boosted PI. During the follow-up, none of the patients changed from ATV_rtv_ to ATV_400_.

### Effectiveness and safety

After a follow-up of 48 weeks, the Kaplan–Meier estimates of effectiveness by intention-to-treat analysis were 85.9% (CI_95_, 80.3–91.4%) and 87.6% (CI_95_, 81.0–94.1%), (p = 0.684) for ATV_rtv_ and ATV_400_ plus 3TC, respectively. The corresponding values obtained by on-treatment analyses were 97.7% (CI_95_, 95.2−100%) and 98.8% (CI_95_, 97.0−100%) (p = 0.546). When comparing the effectiveness in retrospectively and prospectively included patients, no differences were found (data not shown). Overall, three cases of VF occurred in the ATV_rtv_ group (at month 3, 9 and 12) and only one case (at month 6) in the ATV_400_ group_;_ no results for genotype tests were available due to low HIV-RNA levels that impeded amplification. Two of these patients achieved virological suppression three months later; one of them by switching to a triple therapy regimen, and another one while continuing with the ATV dual therapy after adherence counseling. Treatment effectiveness was not affected by sex, presence of chronic hepatitis C, cirrhosis, previous ART, blips, previous VF or treatment group. In univariate analysis, only HIV risk factor was associated to high rate of treatment failure. This factor remained significant when a multivariate analysis was performed (Table 2).

**Table 2:** Univariate and multivariate analyses to identify factors associated with treatment efficacy assessed in an intention-to-treat analysis (n = 246).

Parameter	n	Virological success, n (%)	*p* univariate	AHR (95% CI)	*p* multivariate
Age* (years)					
< 50	122	103 (84.4)	0.324	0.96 (0.92−1.01)	0.148
≥ 50	124	110 (88.7)		1	
BMI					
< 24	91	81 (89)	0.760		
≥ 24	138	121 (87.7)			
Gender					
Female	63	54 (84.1)	0.507	0.57 (0.26−1.25)	0.163
Male	183	160 (87.4)			
Previous iv drug use					
No	145	133 (91.7)	0.005	3.73 (1.50−9.28)	**0.005**
Yes	101	80 (79.2)			
Chronic hepatitis C					
No	146	130 (89)	0.153	1.18 (0.51−2.73)	0.697
Yes	98	81 (82.7)			
Cirrhosis					
No	210	182 (86.7)	0.653		
Yes	29	26 (89.7)			
Previous ART					
PI-based	221	207 (98.1)	0.831		
NNRTI-based	24	24 (100)			
INSTI-based	10	10 (100)			
Previous VF with PI					
No	184	160 (87)	0.989		
Yes	61	53 (86.9)			
Previous blips					
No	198	173 (87.4)	0.678		
Yes	47	40 (85.1)			
Treatment group					
ATV_rtv_	149	128 (85.9)	0.698		
ATV_400_	97	85 (87.6)			

AHR: adjusted hazard ratio; CI: confidence interval; VF: virological failure; PI, protease inhibitor; NRTI, nucleoside reverse transcriptase inhibitor; INSTI, integrase strand transfer inhibitor; ATV_rtv_: ritonavir-boosted atazanavir; ATV_400_: unboosted atazanavir

*entered as continuous variable in the multivariate analysis; Virological success: proportion of patients with plasma HIV-RNA < 20 copies/mL at 48 weeks.

In addition to VF, other treatment failures in the ATV_rtv_ group were due to AEs [grade 3 hyperbilirubinemia (n = 2; 1.3%)], loss to follow-up or voluntary treatment drop-out [n = 9 (6.0%)], and change of treatment to a single-tablet regimen due to medical decision without underlying VF criteria or AEs [n = 7 (4.7%)]. In the group of ATV_400_ the numbers of non-virological failures were 1 (1%) for AEs (grade 1 gastrointestinal disorder), 6 (6.2%) for loss to follow-up, and 4 (4.1%) for change of treatment by medical decision. All patients had an undetectable viral load at the time of the last available HIV-RNA assessment on ATV.

During the 48 weeks of follow-up, 20 (13.4%) patients on ATV_rtv_ experienced blip episodes (1 blip, 17 patients; 2 blips, 3 patients), while only eight (8.2%) patients on ATV_400_ experienced nine blip episodes (p = 0.148), with a median HIV-RNA of 129 copies/mL (IQR, 79–273).

The median increase in CD4^+^ T cell counts from baseline to week 48 was 20 cells/μL (IQR, -80−124) and 50 cells/μL (IQR, -85–133) in the ATV_rtv_ and ATV_400_ groups, respectively, being inversely proportional to the baseline CD4^+^ T cell counts (r = -0.248 and -0.375, respectively; p = 0.006)

Aminotransferase level elevations throughout the follow-up period occurred in 4 patients in the ATV_rtv_ group (grade 1, 2; grade 2, 2), three of them suffered chronic hepatitis C, and in 1 patient in the ATV_400_ group (grade 1), who was also coinfected by hepatitis C virus. These alterations were transient and improved without treatment modification in all cases. Regarding changes in lipid profiles between baseline and week 48, no significant differences between the ATV_rtv_ and ATV_400_ groups were observed, with median changes (mg/dL) of 8 (IQR, -10–28) versus -12 (IQR, -39–22) in fasting total cholesterol, 1 (IQR, -7–7) versus -2 (IQR, -11–1) in HDL-cholesterol, 12 (IQR, -10–34) versus -12 (IQR, -27–9) in LDL-cholesterol, and 4 (IQR, - 45–52) versus -25 (IQR, -62– -1) in triglycerides, respectively.

### Pharmacokinetics of ATV

**Fig 1:**
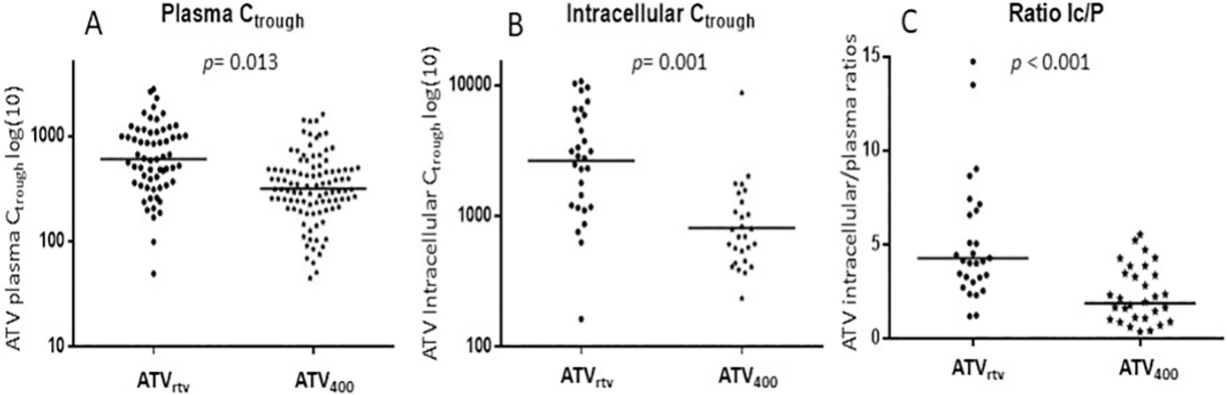
Atazanavir trough concentrations (C_trough_) in plasma (A), intracellular (B), and intracellular/plasma ratios (Ic/P) in patients receiving once-daily ritonavir-boosted atazanavir (300/100 mg) (ATV_rtv_) or unboosted atazanavir (400 mg) (ATV_400_).

The pharmacokinetics study was conducted in a sub-group of 63 and 78 patients in the ATV_rtv_ and ATV_400_ group, respectively. ATV C_trough_ were determined in 339 plasma samples **(**ATV_rtv,_ 108; ATV_400_, 231), and in 35 PBMCs samples of each group (Fig 1). In both plasma and intracellular compartments, the ATV C_trough_ were higher with ATV_rtv_ than with ATV_400._ In plasma samples, the ATV concentrations ranged from 49.6 to 3698.0 ng/mL with a GM of 605.9 ng/mL (IQR, 370.4–1063.3) for ATV_rtv_ and from 45.1 to 1755.0 ng/mL (GM, 318.3 ng/mL; IQR, 218.5–483.4) for ATV_400_ (p <0.001). Whereas in PBMCs samples, the corresponding values were 2659.2 (IQR, 1213.3–5940.7; range 163.7–10743.0) vs. 811.3 ng/mL (IQR497.5–1180.4; range 235.8–8778.6) (p <0.001) for ATV_rtv_ and ATV_400_, respectively. Likewise, the mean intracellular penetration, evaluated as intracellular/plasma C_trough_ ratios was higher in the ATV_rtv_ group than in the ATV_400_ group (4.39 vs. 2.54; p <0.001). There was a correlation between the plasma and intracellular C_trough_ in patients taking ritonavir-boosted ATV (r = 0.754, p < 0.001), but not in those on unboosted ATV (r = 0.252, p = 0.123).

ATV concentrations below the suggested minimum effective concentration of 150 ng/mL were detected in two (1.8%) samples from 2 patients of the ATV_rtv_ group and 43 (17.7%) samples from 12 patients of the ATV_400_ group. Given the low number of VF, it was not possible to establish any relationship between VF and plasma or intracellular concentrations, however, none of the patients with plasma ATV C_trough_ below 150 ng/mL had VF. No association between blip episodes and a low ATV C_trough_ was observed. Likewise, there were no relationships between plasma and intracellular ATV concentrations and weight or body mass index, and there were no differences in plasma and intracellular ATV concentrations with respect to gender or the presence of cirrhosis.

For the ATV_rtv_ and ATV_400_ groups, the median intra-subject variability for ATV was 32.3% and 44.6% in plasma samples and 34.5% and 40.3% for intracellular ATV. The corresponding median inter-subject variability was 197.23% and 99.23% in plasma, and 118.8% and 97.2% for cellular samples.

## Discussion

In the last years, several ART simplification strategies have been studied in order to reduce the severity or even avoid AEs from chronic drug exposure, lower the risk of HIV-1 drug resistance, and achieve more cost-effective regimens. Among them are dual therapies based on ritonavir-boosted PIs in combination with 3TC or other drugs that have shown good efficacy rates and safety profiles, being this simplified regimen an option in low resource settings or when clinicians prefer the least number of drugs possible for their patients. [11–13, 28–30]. ATV is the only HIV-1 protease inhibitor currently in use that can be administered without a pharmacokinetic enhancer, although a dose increase from 300 to 400 mg daily is recommended for the unboosted regimen. Apart from the prevention of potential drug-drug interactions, this adaptability enables to manage both ritonavir-induced toxicity and hyperbilirubinemia caused by high ATV concentrations.

In spite of being a study performed in the routine clinical practice, a high effectiveness of ATV plus 3TC was observed at 48 weeks, although it should be taken into account that most of patients of the study were virologically suppressed for a long time. Furthermore, less than 2% of the population presented AEs that led to treatment discontinuation, which may partly be explained by the large proportion of patients who were already on an ATV_rtv_-based regimen before switching to dual therapy. Our results are similar to those reported by the ATLAS-M clinical trials [12], where the efficacy rates at week 48 by intention-to-treat and on-treatment analyses were 89.5% (CI_95_, 84.3–94.7%) and 90.1% (CI_95_, 85.0–95.2%) respectively, and even better than those in the SALT trial [13] that found an efficacy rates of 78% and 83%. Both trials demonstrated the non-inferiority of this combination compared to ATV_rtv_  +  2 plus two NRTIs in long-term supressed patients. To our knowledge, only two studies of dual therapy comprising ATV_400_ plus 3TC [19–20] are available. In one, patients with virological suppression on ATV_400_ plus two NRTIs were switched to ATV_400_ plus 3TC or emtricitabine, without any VF or discontinuation after 48 weeks. In the other study, treatment-experienced HIV-infected patients were switched from triple therapy to boosted or unboosted ATV plus 3TC with no VF. However, the latter study only analyzed the first six months after switching to dual therapy, and sample sizes were as low as 40 and 20 patients, respectively.

In accordance with previous pharmacokinetic studies, the herein presented work finds lower ATV C_trough_ in the non-boosted regimen as compared with the ritonavir-boosted regimen both in plasma and on intracellular level [31–33]. The underlying mechanism is likely the potent inhibitory effect of ritonavir on P-glycoprotein, of which atazanavir is substrate, thus facilitating a better absorption and the accumulation of the drug on the intracellular level [34–35]. In the ATV expanded access program, Gonzalez de Requena *et al*. [21] found that an ATV C_trough_ lower than 150 ng/mL was associated with a high probability of VF in PI-experienced patients, most of them showing plasma HIV-RNA loads higher than 1000 copies/mL. In contrast, none of the patients with a plasma ATV C_trough_ below 150 ng/mL had VF in the present study, although it is to note that the clinical condition of the herein analyzed populations was different regarding their virological and immunologic status.

One of the limitations of this study is that in the last years the use of ATV_rtv_ is progressively decreasing since it is being replaced by a co-formulation including the pharmacokinetic enhancer cobicistat, which is not analyzed in the present study. However, two randomized, crossover bioequivalence studies have shown that ATV/cobicistat (300/150 mg) provides bioequivalent ATV exposures as compared to ATV_rtv,_ both in healthy volunteers and treatment-naïve adults infected with HIV-1 [36–37]. Although cobicistat shows a better tolerance and drug-drug interaction profile than ritonavir, in some settings it should be avoided and ATV_400_ may be a good alternative. Another limitation is the low number of blood samples for pharmacokinetics study, since this substudy was only conducted in the prospective part. However, the determination of ATV plama levels was not the primary aim of the study and furthermore, still a considerably number of samples were obtained.

In conclusion, our data suggest that boosted or unboosted atazanavir plus lamivudine represent comparable simplification strategies regarding effectiveness and safety in HIV-infected patients with long-term virological suppression. Although lower plasma and intracellular ATV C_trough_ is observed with ATV_400_, this does not appear to compromise the effectiveness of these regimens in virologically suppressed patients. However, the lasting efficacy of this regimen would need further investigation with clinical trials.

## Supporting information

S1 Table Baseline characteristic according to period of inclusion and treatment group. M (IQR), Mean (interquartile range). ATVrtv, atazanavir boosted with ritonavir. ATV400, unboosted-atazanavir. ART, antiretroviral treatment. PIrtv, ritonavir-boosted protease inhibitor. NRTIs, nucleoside reverse transcriptase inhibitors. INSTI, integrase strand transfer inhibitor.
(DOCX)
