# Supplementary material for: No difference in effectiveness of treatment simplification to boosted or unboosted atazanavir plus lamivudine in virologically suppressed in HIV-1-infected patients
Source: PLoS One. 2018 Sep 20;13(9):e0203452. doi: 10.1371/journal.pone.0203452 (PMC6147473; doi:10.1371/journal.pone.0203452)
Supplement: S1 Table — M (IQR), Mean (interquartile range). ATVrtv, atazanavir boosted with ritonavir. ATV400, unboosted-atazanavir. ART, antiretroviral treatment. PIrtv, ritonavir-boosted protease inhibitor. NRTIs, nucleoside reverse transcriptase inhibitors. INSTI, integrase strand transfer inhibitor. (DOCX) [file pone.0203452.s001.docx]

**S1 Table. Baseline Characteristic according to period of inclusion and treatment group.**

|  | **ATV_rtv_ + 3TC** | | | **ATV_400_ + 3TC** | | |
| --- | --- | --- | --- | --- | --- | --- |
|  | Retrospectively n= 67 | Prospectively n= 82 | ***p*** | Retrospectively n= 19 | Prospectively n= 78 | ***p*** |
| Male, no. (%) | 48 (71.6) | 65 (79.3) | 0.288 | 11 (57.9) | 59 (75.6) | 0.172 |
| Age, years, M (IQR) | 50 (41-54) | 48 (43-52) | 0.860 | 54 (46-62) | 51 (45-54) | 0.117 |
| Weight, kg, M (IQR) | 69 (59-78) | 72 (67-81) | 0.080 | 71 (60-92) | 70 (65-80) | 0.803 |
| Nadir CD4^+^/µl, M (IQR) | 180 (66-296) | 147 (53-245) | 0.076 | 227 (130-250) | 154 (59-307) | 0.412 |
| Risk factor for HIV, no. (%) |  |  | 0.093 |  |  | 0.122 |
| Previous iv drug use | 23 (34.3) | 40 (48.8) |  | 5 (26.3) | 34 (43.6) |  |
| Homosexual | 23 (34.3) | 23 (28.0) |  | 4 (21.1) | 17 (21.8) |  |
| Heterosexual | 21 (31.3) | 19 (23.2) |  | 10 (52.6) | 27 (28.3) |  |
| Chronic hepatitis C, no. (%) | 21 (31.3) | 34 (41.5) | 0.185 | 10 (52.6) | 33 (42.2) | 0.313 |
| Cirrhosis no. (%) | 7 (10.4) | 11 (13.4) | 0.572 | 2 (10.5) | 9 (11.5) | 0.874 |
| Previous months on ART, M (IQR) | 40 (23-119) | 139 (64-200) | 0.000 | 48 (23-59) | 72 (36-121) | 0.304 |
| Months with HIV-RNA < 20 copies/mL, M (IQR) | 60 (36-108) | 60 (33-121) | 0.505 | 84 (54-121) | 84 (48-120) | 0.757 |
| Previous ART combinations |  |  | 0.000 |  |  | 0.221 |
| PI_rtv_ (+ 2 NRTIs), n (%) | 46 (68.7) | 37 (45.1) |  | 15 (78.9) | 48 (61.5) |  |
| Atazanavir | 39 (84.8) | 34 (91.9) |  | 15 (100) | 46 (95.8) |  |
| Darunavir | 3 (6.5) | 0 (0.0) |  | 0 (0.0) | 2 (4.8) |  |
| Lopinavir | 4 (8.7) | 3 (8.1) |  | 0 (0.0) | 0 (0.0) |  |
| NNRTI (+ 2 NRTIs), n (%) | 10 (14.9) | 6 (7.3) |  | 1 (5.3) | 7 (9.0) |  |
| Efavirenz | 4 (60) | 2 (33.3) |  | 1 (100) | 3 (42.9) |  |
| Nevirapine | 0 (0.0) | 1 (16.7) |  | 0 (0.0) | 1 (14.3) |  |
| Rilpivirine | 6(40) | 3 (50) |  | 0 (0.0) | 3 (42.9) |  |
| INSTI (+ 2 NRTIs), n (%) | 1 (1.5) | 5 (6.1) |  | 0 (0.0) | 4 (5.1) |  |
| Elvitegravir | 0 (0.0) | 1 (20) |  | - | 1 (25) |  |
| Dolutegravir | 0 (0.0) | 0 (0.0) |  | - | 1 (25) |  |
| Raltegravir | 1 (100) | 4 (80) |  | - | 2 (50) |  |
| Other PIrtv regimens, n (%) | 10 (14.9) | 34 (41.5) |  | 3 (15.8) | 19 (24.4) |  |
| CD4^+^/µl, M (IQR) | 644 (520-886) | 728 (510-945) | 0.356 | 612 (384-712) | 713 (562-961) | 0.308 |

**S1 Table .** M (IQR), Mean (interquartile range). ATV_rtv_, atazanavir boosted with ritonavir. ATV_400_, unboosted-atazanavir. ART, antiretroviral treatment. PI_rtv_, ritonavir-boosted protease inhibitor. NRTIs, nucleoside reverse transcriptase inhibitors. INSTI, integrase strand transfer inhibitor.
